# Using artificial intelligence to predict adverse outcomes in emergency department patients with hyperglycemic crises in real time

**DOI:** 10.1186/s12902-023-01437-9

**Published:** 2023-10-24

**Authors:** Chin-Chuan Hsu, Yuan Kao, Chien-Chin Hsu, Chia-Jung Chen, Shu-Lien Hsu, Tzu-Lan Liu, Hung-Jung Lin, Jhi-Joung Wang, Chung-Feng Liu, Chien-Cheng Huang

**Affiliations:** 1 Department of Emergency Medicine, Chi Mei Medical Center, 901 Zhonghua Road, Yongkang District, Tainan City, 710 Taiwan; 2 Graduate Institute of Medical Sciences, College of Health Sciences, Chang Jung Christian University, Tainan, Taiwan; 3 School of Medicine, College of Medicine, National Sun Yat-sen university, Kaohsiung, Taiwan; 4 Information Systems, Chi Mei Medical Center, Tainan, Taiwan; 5 Department of Nursing, Chi Mei Medical Center, Tainan, Taiwan; 6 Department of Emergency Medicine, Taipei Medical University, Taipei, Taiwan; 7 Department of Anesthesiology, Chi Mei Medical Center, Tainan, Taiwan; 8 Department of Anesthesiology, National Defense Medical Center, Taipei, Taiwan; 9 Department of Medical Research, Chi Mei Medical Center, 901 Zhonghua Road, Yongkang District, Tainan City, 710 Taiwan; 10 Department of Emergency Medicine, Kaohsiung Medical University, Kaohsiung, Taiwan; 11 Department of Environmental and Occupational Health, College of Medicine, National Cheng Kung University, Tainan, Taiwan

**Keywords:** Adverse outcome, Artificial intelligence, Emergency department, Hyperglycemic crises, Intensive care unit, Machine learning, Mortality, Multilayer perceptron, Sepsis

## Abstract

**Background:**

Hyperglycemic crises are associated with high morbidity and mortality. Previous studies have proposed methods to predict adverse outcomes of patients in hyperglycemic crises; however, artificial intelligence (AI) has never been used to predict adverse outcomes. We implemented an AI model integrated with the hospital information system (HIS) to clarify whether AI could predict adverse outcomes.

**Methods:**

We included 2,666 patients with hyperglycemic crises from emergency departments (ED) between 2009 and 2018. The patients were randomized into a 70%/30% split for AI model training and testing. Twenty-two feature variables from the electronic medical records were collected. The performance of the multilayer perceptron (MLP), logistic regression, random forest, Light Gradient Boosting Machine (LightGBM), support vector machine (SVM), and K-nearest neighbor (KNN) algorithms was compared. We selected the best algorithm to construct an AI model to predict sepsis or septic shock, intensive care unit (ICU) admission, and all-cause mortality within 1 month. The outcomes between the non-AI and AI groups were compared after implementing the HIS and predicting the hyperglycemic crisis death (PHD) score.

**Results:**

The MLP had the best performance in predicting the three adverse outcomes, compared with the random forest, logistic regression, SVM, KNN, and LightGBM models. The areas under the curves (AUCs) using the MLP model were 0.852 for sepsis or septic shock, 0.743 for ICU admission, and 0.796 for all-cause mortality. Furthermore, we integrated the AI predictive model with the HIS to assist decision making in real time. No significant differences in ICU admission or all-cause mortality were detected between the non-AI and AI groups. The AI model performed better than the PHD score for predicting all-cause mortality (AUC 0.796 vs. 0.693).

**Conclusions:**

A real-time AI predictive model is a promising method for predicting adverse outcomes in ED patients with hyperglycemic crises. Further studies recruiting more patients are warranted.

**Supplementary Information:**

The online version contains supplementary material available at 10.1186/s12902-023-01437-9.

## Background

Diabetic ketoacidosis (DKA) and hyperosmolar hyperglycemic state (HHS) are severe acute complications of diabetes [1]. Precipitating factors include uncontrolled type 1 and 2 diabetes, infection, new-onset diabetes, pancreatitis, acute coronary syndrome, stroke, and medications [2, 3]. Visits to the emergency department (ED) for DKA and HHS have been increasing annually in the United States. In 2015, there were 3.1 visits for DKA and 2.9 visits for HHS per 10,000 adults with diabetes [1]. Although treatment includes hydration, insulin therapy, and electrolyte replacement, the mortality rate for hyperglycemic crises remains high [4, 5] and can also increase the risk for subsequent adverse cardiovascular events, end-stage renal disease, and long-term mortality [6–8]. Risk stratification (e.g., sepsis, intensive care unit [ICU] admission, and mortality) may improve outcomes in hyperglycemic crises [2, 3]. Prior studies identified mortality predictors, such as age, mental status, severe coexisting diseases, serum pH < 7.0, high insulin dose within the first 12 h, and serum glucose > 16.7 mmol after 12 h [4, 5, 8], but a clinical prediction rule may be more practical.

In 2013, the predicting the hyperglycemic crisis death (PHD) score was proposed as a tool to help ED physicians stratify the mortality risk and make decisions regarding patients in hyperglycemic crises [7]. It consists of six predictors and stratifies patients into low, intermediate, and high-risk groups. While the area under the curve (AUC) for the rule was 0.925 in the validation set, the PHD score was limited by a small derivation sample and manual calculation [7]. In recent years, artificial intelligence (AI) techniques have become a promising method to assist in medical decisions, and several AI predictions for adverse outcomes have been implemented in ED [6, 9–11]. However, no study has yet evaluated the feasibility and accuracy of AI predictions of adverse outcomes in ED patients with hyperglycemic crises in real time [12, 13]. Therefore, we carried out this study to clarify it.

## Methods

### Study design, setting, and participants

**Fig. 1:**
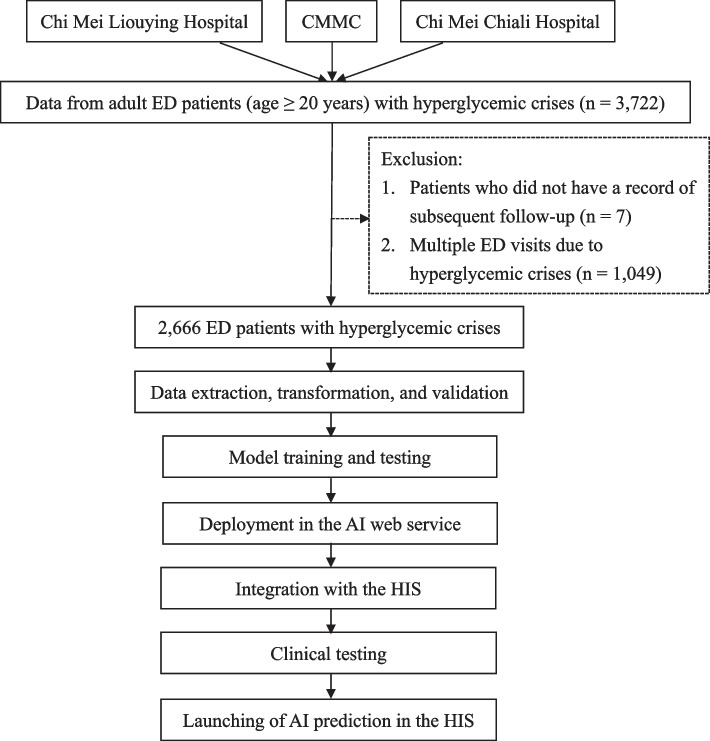
Study flow chart. CMMC, Chi Mei Medical Center; ED, emergency department; AI, artificial intelligence; HIS, hospital information system

We established a multi-disciplinary team at the Chi Mei Medical Center (CMMC), including emergency physicians, data scientists, information engineers, nurse practitioners, and quality managers to implement big data and AI. Adults (age ≥ 20 years) with hyperglycemic crises who visited the EDs of three hospitals (CMMC, Chi Mei Liouying Hospital, and Chi Mei Chiali Hospital) between 2009 and 2018 were recruited (Fig. 1). The rationale that we used to select patients aged ≥ 20 years is that a criterion for an adult in Taiwan is “ ≥ 20 years”, and it has been adopted in many studies [6, 11]. The criteria for hyperglycemic crises were defined as the final diagnosis of DKA or HHS in the ED, using the International Classification of Diseases, Ninth Revision, Clinical Modification (ICD-9-CM) codes 250.1 or 250.2 and ICD-10 codes E11.1 or E11.0. Patients who did not have a record of subsequent follow-up and those who visited the ED for multiple hyperglycemic crises were excluded.

### Definition of feature variables

The 22 feature variables retained for analysis were age, sex, body mass index (BMI), vital signs at triage (Glasgow Coma Scale [GCS], systolic blood pressure, heart rate, respiratory rate, and body temperature), bedridden, nasogastric tube feeding, history of hypertension (ICD-9-CM: 401–405 or ICD-10: I10–I16), hyperlipidemia (ICD-9-CM: 272.0–272.5, 277.7 or ICD-10: E78.0–E78.5, E88.81), malignancy (ICD-9-CM: 140–208 or ICD-10: C00–C69), chronic kidney disease (ICD-9-CM: 585 or ICD-10: N18), and laboratory data, including blood urea nitrogen, serum creatinine, white blood cell count, hemoglobin, glucose, and high sensitive C-reactive protein (hs-CRP), as well as concomitant infection (ICD-9-CM: 001–139, 320–326, 390–392, 480–488, 540–543, 555–558, 566–567, 599.0, 601, 604, 614–616, 680–686, 730 or ICD-10: A00–B99, G00–G09, I00–I02, J09–J18, K35–K38, K50–K52, K61, K65, N39.0, N41, N45, N70–N77, L00–L08, M86, R65). The feature variables were suggested predictors of adverse outcomes in previous studies, and possible risk factors for adverse outcomes in clinical practice [7, 14–17]. History was pre-existing at the time of presentation as diagnosed by the physician in the electronic medical records (EMRs). Age was divided into four subgroups of 20–34, 35–49, 50–64, 64–74, and ≥ 75 years according to previous studies [6, 11, 18]. BMI was divided into four subgroups according to the Asian BMI levels: < 18.5, 18.5–22.9, 23–24.9, and ≥ 25 kg/m^2^ [19, 20].

### Outcome measurements

We defined three adverse outcomes, including sepsis or septic shock < 1 month (ICD-9-CM: 038, 790.7 or ICD-10: A40–A41, R65, R7881), ICU admission < 1 month, and all-cause mortality < 1 month following the time of presentation in the ED. The general “ICU admission” criteria in the study hospital were unstable vital signs and the need for intensive monitoring and treatment. “All-cause mortality” was defined as a record of death certification or discharge against medical advice in a patient in critical condition in the EMRs. We defined “ < 1 month” for outcomes according to previous studies of hyperglycemic crises and AI [7, 11].

### Ethical statement

This study was approved by the Institutional Review Board of the CMMC and was conducted according to the Declaration of Helsinki. Informed consent from the patients was waived because this study was retrospective and contained de-identified information, which did not affect the rights or welfare of the patients.

### Data processing, comparison, and application

The study had two phases: pre- and post-implementation. The pre-implementation phase developed an AI predictive model and integrated it with the HIS. The post-implementation phase compared outcomes between the non-AI and AI groups. The feature of sex was transformed into 1 (male) or 0 (female). Missing or ambiguous data were defined by a team comprising emergency physicians, data scientists, information engineers, nurse practitioners, and quality managers. Data with missing feature variables were deleted or estimated with an average value. Second, we divided the data into training (70%) and test (30%) datasets according to previous studies [6, 11, 21]. There were fewer outcomes, particularly ICU admissions, which may have caused an imbalance in the data. Therefore, we used the synthetic minority over-sampling technique to improve the data imbalance in the training dataset [22]. Machine learning (ML) and deep learning (DL) are the two major fields of AI [23]. ML, including random forest, logistic regression, support vector machine (SVM), K-nearest neighbor (KNN), and Light Gradient Boosting Machine (LightGBM), is the ability that a computer system uses to automatically improve their function or to “learn” with continuous data [23]. DL, as the multilayer perceptron (MLP) in this study, has a more complex network of nodes between the inputs and outputs for solving complex problems more accurately [23]. Because the case number was small, we used MLP, a classical neural network method, to represent the DL method. The MLP has been adopted successfully in our studies [6, 9, 11, 24, 25]. We used fivefold cross validation technique to build all models. We compared the ML algorithms, including random forest, logistic regression, SVM, KNN, LightGBM, and MLP for accuracy, sensitivity, specificity, positive predictive value (PPV), negative predictive value (NPV), F1, and AUC. Accuracy was defined as the fraction of cases that the model correctly predicted [26]. Sensitivity was the fraction of positive cases predicted as positive [26]. Specificity was the fraction of negative cases predicted as negative [26]. PPV was the fraction of true positive cases from all cases that the model predicted to be positive [26]. NPV was the fraction of negative cases from all cases that the model predicted to be negative [26]. F1 was the harmonic mean of PPV and sensitivity [26]. Accuracy, PPV, NPV, and F1 depend on the prevalence of adverse outcomes [26]. We used the AUC to determine the best model for further implementation [13–15] because the AUC considers the predictive performance of the positive and negative outcomes. An AUC of 0.5 suggests no discrimination, 0.7–0.8 suggests acceptable, 0.8–0.9 suggests excellent, and > 0.9 suggests outstanding [26]. The tuning parameters we used to refine our models are shown in Supplementary Table 1. We performed the DeLong test to assess overfitting of the training and test models and plotted the learning curves for our model (best model) [27]. The *p*-value of the DeLong test for the best model (MLP model) was > 0.05, indicating no significant difference between the training and test models. Therefore, no significant overfitting existed. Using the learning curve [28] (Supplementary Fig. 1), we observed no significant overfitting as the number of samples increased, with the training score (F1 score) curve gradually approaching and overlapping the testing score curve. Subsequently, we integrated the AI predictive model into the HIS, deployed it at the AI web service, and launched it for real-time decision-making assistance by ED physicians. To reveal the real-time prediction result, a physician simply needed to press the AI button set up in the HIS. We then conducted a retrospective impact study between December 1, 2019, and April 30, 2021, in which all ED patients with hyperglycemic crises were identified and divided into non-AI and AI groups to compare outcomes. The use of AI was an aid to decision-making and depended on the physician's discretion.

### ML algorithms used in this study

MLP is an artificial neural network that maps input data to appropriate outputs using an input layer, hidden layer, and output layer, each connected by a synaptic weight matrix and with nonlinear activation functions and trained via backpropagation [29]. Its multiple layers and activation functions enable it to distinguish non-linearly separable data [29]. In a study predicting adverse outcomes from pneumonia, MLP had AUCs of 0.749, 0.792, and 0.802 for sepsis or septic shock, respiratory failure, and mortality, respectively [6].

Random forest is an efficient ensemble technique that contains multiple decision trees generated from combined optimization decision trees, useful for classification and regression, and preventing overfitting with high accuracy even for incomplete datasets [30]. Random forest has been widely used in AI medical studies for prediction [31], including a study of predicting outcomes in older ED patients with influenza, where their random forest model achieved an AUC of 0.840 for hospitalization, 0.765 for pneumonia, 0.857 for sepsis or septic shock, 0.885 for ICU admission, and 0.875 for in-hospital mortality [9].

Logistic regression is a statistical approach and supervised ML algorithm used for classification problems by mapping features to categorical targets and predicting the probability of a new case belonging to a target class [32]. In a recent study of predicting major adverse cardiac events in ED patients with chest pain, logistic regression was used to achieve AUCs of 0.868 for acute myocardial infarction at < 1 month and 0.716 for all-cause mortality at < 1 month [11].

LightGBM is a high-performing gradient boosting framework that utilizes tree-based learning algorithms and includes Gradient-based One-Side Sampling and Exclusive Feature Bundling methods for selective sampling and reduced dimensionality [33]. A study using LightGBM as an algorithm reported AUCs of 0.774 for sepsis or septic shock, 0.847 for respiratory failure, and 0.835 for mortality prediction [6].

SVM is a versatile algorithm that can address regression, binary, and multi-class classification problems by identifying a hyperplane that maximizes the distance between classes in the feature space [34]. In cases where the classes are not linearly separable, the kernel trick is used to project the feature vectors to a higher-dimensional space [34]. SVM is widely used in medicine, with a study reporting AUCs of 0.840 for hospitalization, 0.733 for pneumonia, 0.806 for sepsis or septic shock, 0.778 for ICU admission, and 0.762 for in-hospital mortality in older patients with influenza [9].

KNN is a non-parametric, supervised learning classifier that predicts the grouping of an individual data point using proximity to other data points [35]. A study using KNN to predict major adverse cardiac events in ED patients with chest pain reported AUCs for acute myocardial infarction at < 1 month and all-cause mortality at < 1 month of 0.865 and 0.969, respectively [11].

## Results

A total of 2,666 ED patients with hyperglycemic crises were recruited into the study at the three hospitals between 2009 and 2018 (Table 1). Their mean age was 65.3 ± 16.9 years, and the percentage of females was 45.7%. The four age subgroups were 20–34 years (5.8%), 35–49 years (11.9%), 50–64 years (25.8%), 65–74 years (20.2%), and ≥ 75 years (36.3%). The mean BMI was 23.0 ± 4.8 kg/m^2^. There were 60.2% of bedridden patients and 8.0% of patients being fed by nasogastric tube. A history of hypertension (53.0%), hyperlipidemia (26.2%), cerebrovascular accident (29.8%), malignancy (14.2%), and chronic kidney disease (11.4%) were found. Concomitant infection was found in 46.8% of the patients. Within 1 month, 31.7% of patients had sepsis or septic shock, 6.0% required ICU admission, and 12.8% died. Missing data were assigned values according to decisions made at a multi-disciplinary team meeting (Supplementary Table 2).

**Table 1:** Characteristics of all ED patients with hyperglycemic crises in the three hospitals

	Total	Sepsis or septic shock			ICU admission			All-cause mortality		
		No	Yes	*p*-value	No	Yes	*p*-value	No	Yes	*p*-value
Number of patients	2666 (100)	1820 (68.3)	846 (31.7)		2505 (94.0)	161 (6.0)		2326 (87.2)	340 (12.8)	
Age (years)	65.3 ± 16.9	63.7 ± 17.5	68.9 ± 14.9	< 0.001	65.3 ± 16.9	65.8 ± 16.1	0.685	64.3 ± 17.0	72.6 ± 13.8	< 0.001
Age subgroup				< 0.001			0.646			< 0.001
20 − 34 years	154 (5.8)	135 (7.4)	19 (2.2)		146 (5.8)	8 (5.0)		152 (6.5)	2 (0.6)	
35 − 49 years	317 (11.9)	245 (13.5)	72 (8.5)		302 (12.1)	15 (9.3)		300 (12.9)	17 (5.0)	
50 − 64 years	689 (25.8)	481 (26.4)	208 (24.6)		642 (25.6)	47 (29.2)		619 (26.6)	70 (20.6)	
65 − 74 years	539 (20.2)	354 (19.5)	185 (21.9)		503 (20.1)	36 (22.4)		462 (19.9)	77 (22.6)	
≥ 75 years	967 (36.3)	605 (33.2)	362 (42.8)		912 (36.4)	55 (34.2)		793 (34.1)	174 (51.2)	
Sex				0.010			0.323			0.573
Female	1218 (45.7)	800 (44.0)	418 (49.4)		1151 (45.9)	67 (41.6)		1068 (45.9)	150 (44.1)	
Male	1448 (54.3)	1020 (56.0)	428 (50.6)		1354 (54.1)	94 (58.4)		1258 (54.1)	190 (55.9)	
BMI (kg/m^2^)	23.0 ± 4.8	23.3 ± 4.9	22.5 ± 4.5	< 0.001	23.0 ± 4.7	23.5 ± 5.2	0.286	23.2 ± 4.8	21.6 ± 4.3	< 0.001
Asian BMI level subgroup				0.011			0.526			< 0.001
< 18.5	327 (15.0)	202 (13.9)	125 (17.5)		302 (14.9)	25 (17.1)		267 (13.9)	60 (23.3)	
18.5 − 22.9	866 (39.9)	564 (38.7)	302 (42.2)		815 (40.2)	51 (34.9)		754 (39.4)	112 (43.6)	
23 − 24.9	353 (16.2)	246 (16.9)	107 (14.9)		325 (16.0)	28 (19.2)		310 (16.2)	43 (16.7)	
≥ 25	627 (28.9)	445 (30.5)	182 (25.4)		585 (28.9)	42 (28.8)		585 (30.5)	42 (16.3)	
Vital signs at triage										
Glasgow coma scale	12.4 ± 3.8	13.0 ± 3.4	11.0 ± 4.1	< 0.001	12.5 ± 3.7	10.5 ± 4.7	< 0.001	12.8 ± 3.5	9.6 ± 4.3	< 0.001
Systolic blood pressure (mmHg)	137.6 ± 34.7	140.3 ± 33.8	131.9 ± 35.8	< 0.001	138.5 ± 34.2	123.2 ± 38.3	< 0.001	139.5 ± 33.7	124.0 ± 38.1	< 0.001
Heart rate (beats/min)	105.9 ± 23.8	103.2 ± 23.0	111.8 ± 24.2	< 0.001	105.5 ± 23.6	112.9 ± 25.6	0.001	105.2 ± 23.2	110.6 ± 27.0	0.001
Respiratory rate (breaths/min)	20.2 ± 4.4	19.7 ± 3.9	21.2 ± 5.2	< 0.001	20.0 ± 4.2	21.9 ± 6.1	< 0.001	19.8 ± 4.0	22.3 ± 6.1	< 0.001
Body temperature (°C)	36.7 ± 0.9	36.6 ± 0.7	37.0 ± 1.1	< 0.001	36.7 ± 0.9	36.7 ± 0.9	0.940	36.7 ± 0.9	36.8 ± 1.0	0.051
Bedridden	1604 (60.2)	963 (52.9)	641 (75.8)	< 0.001	1475 (58.9)	129 (80.1)	< 0.001	1306 (56.1)	298 (87.6)	< 0.001
Nasogastric tub feeding	214 (8.0)	85 (4.7)	129 (15.2)	< 0.001	177 (7.1)	37 (23.0)	< 0.001	156 (6.7)	58 (17.1)	< 0.001
Past histories										
Hypertension	1414 (53.0)	931 (51.2)	483 (57.1)	0.005	1340 (53.5)	74 (46.0)	0.076	1196 (51.4)	218 (64.1)	< 0.001
Hyperlipidemia	698 (26.2)	479 (26.3)	219 (25.9)	0.850	668 (26.7)	30 (18.6)	0.031	607 (26.1)	91 (26.8)	0.845
Cerebrovascular accident	795 (29.8)	486 (26.7)	309 (36.5)	< 0.001	749 (29.9)	46 (28.6)	0.788	664 (28.5)	131 (38.5)	< 0.001
Malignancy	379 (14.2)	252 (13.8)	127 (15.0)	0.458	349 (13.9)	30 (18.6)	0.124	276 (11.9)	103 (30.3)	< 0.001
Chronic kidney disease	305 (11.4)	198 (10.9)	107 (12.6)	0.204	283 (11.3)	22 (13.7)	0.431	258 (11.1)	47 (13.8)	0.165
Laboratory data										
Blood urea nitrogen (mg/dL)	48.7 (34.8)	44.8 (33.2)	56.5 (36.6)	< 0.001	47.4 (33.6)	68.4 (45.1)	< 0.001	45.7 (32.2)	68.7 (43.6)	< 0.001
White blood cell count (103/µL)	11.7 ± 6.0	10.9 ± 5.2	13.2 ± 7.2	< 0.001	11.5 ± 5.9	13.7 ± 7.5	< 0.001	11.8 ± 6.0	10.9 ± 6.0	0.016
Serum creatinine (mg/dL)	1.9 ± 1.6	1.9 ± 1.6	2.0 ± 1.6	0.052	1.9 ± 1.6	2.2 ± 1.5	0.012	1.9 ± 1.6	2.0 ± 1.4	0.406
Hemoglobin (g/dL)	12.8 ± 2.8	13.1 ± 2.8	12.3 ± 2.7	< 0.001	12.8 ± 2.8	12.4 ± 2.9	0.039	13.0 ± 2.8	11.8 ± 2.8	< 0.001
Glucose (mg/dL)	430.7 ± 318.5	433.8 ± 313.9	424.1 ± 328.2	0.473	424.8 ± 309.9	521.2 ± 420.9	0.005	437.5 ± 315.8	384.2 ± 332.8	0.006
hs-CRP (mg/L)	60.6 ± 84.4	39.7 ± 62.4	98.7 ± 103.9	< 0.001	57.9 ± 81.8	98.2 ± 108.0	< 0.001	58.5 ± 84.3	73.3 ± 83.8	0.004
Concomitant infection,	1247 (46.8)	539 (29.6)	708 (83.7)	< 0.001	1145 (45.7)	102 (63.4)	< 0.001	1021 (43.9)	226 (66.5)	< 0.001
PHD score	1.9 ± 1.4	1.5 ± 1.3	2.8 ± 1.1	< 0.001	1.9 ± 1.4	2.5 ± 1.5	< 0.001	1.8 ± 1.3	2.9 ± 1.3	< 0.001
PHD risk class				< 0.001			< 0.001			< 0.001
Low risk (Score 0–2)	1534 (65.0)	1224 (75.8)	310 (41.6)		1466 (66.0)	68 (48.6)		1430 (69.0)	104 (36.1)	
Intermediate risk (Score 3)	531 (22.5)	271 (16.8)	260 (34.9)		491 (22.1)	40 (28.6)		431 (20.8)	100 (34.7)	
High risk (Score ≥ 4)	296 (12.5)	120 (7.4)	176 (23.6)		264 (11.9)	32 (22.9)		212 (10.2)	84 (29.2)	

Data are presented as n (%) or mean ± SD. The independent t-test was used to analyze continuous variables, while the Chi-Square test was utilized to examine categorical variables

*ED* Emergency department, *ICU* Intensive care unit, *BMI* Body mass index, *hs-CRP PHD* Predicting the hyperglycemic crisis death, *SD* Standard deviation

The MLP model outperformed other algorithms with AUCs of 0.852 for sepsis or septic shock, 0.743 for ICU admission, and 0.796 for all-cause mortality in the testing dataset (Table 2 and Supplementary Fig. 2) [36]. After a consensus was reached, MLP was chosen for AI implementation. SHapley Additive exPlanations (SHAP) values were used to identify feature associations and importance (Supplementary Fig. 3). A model was developed for predicting ICU admissions < 48 h with an AUC of 0.780 in the test dataset, outperforming other algorithms. A DeLong test was used to compare AUC values between algorithms (Supplementary Table 4).

**Table 2:** Comparison of performance among the random forest, logistic regression, SVM, KNN, LightGBM, and MLP algorithms for adverse outcomes in ED patients with hyperglycemic crises

Outcomes and algorithms	Accuracy		Sensitivity		Specificity		PPV		NPV		F1		AUC (95%CI)		*p*-value*
	Train	Test	Train	Test	Train	Test	Train	Test	Train	Test	Train	Test	Train	Test	
Sepsis or septic shock															
MLP	0.789	0.790	0.828	0.791	0.750	0.789	0.768	0.636	0.814	0.890	0.797	0.705	0.854 (0.839–0.869)	0.852 (0.825–0.880)	0.910
Random forest	0.920	0.779	0.948	0.780	0.892	0.778	0.898	0.621	0.945	0.884	0.922	0.691	0.984 (0.980–0.987)	0.848 (0.821–0.875)	< 0.001
LightGBM	0.868	0.764	0.896	0.803	0.840	0.745	0.848	0.595	0.889	0.891	0.871	0.683	0.948 (0.940–0.956)	0.842 (0.815–0.870)	< 0.001
SVM	0.855	0.765	0.907	0.764	0.803	0.766	0.821	0.602	0.896	0.874	0.862	0.674	0.945 (0.937–0.953)	0.818 (0.787–0.849)	< 0.001
KNN	0.800	0.738	0.889	0.744	0.711	0.734	0.755	0.566	0.865	0.861	0.817	0.643	0.890 (0.877–0.902)	0.816 (0.786–0.846)	< 0.001
Logistic regression	0.718	0.720	0.690	0.720	0.746	0.720	0.731	0.545	0.706	0.847	0.710	0.620	0.789 (0.771–0.806)	0.802 (0.770–0.833)	0.487
ICU admission															
MLP	0.692	0.680	0.714	0.688	0.670	0.680	0.684	0.120	0.700	0.971	0.698	0.205	0.744 (0.728–0.760)	0.743 (0.663–0.822)	0.973
LightGBM	0.960	0.676	0.924	0.667	0.997	0.677	0.997	0.116	0.929	0.970	0.959	0.198	0.985 (0.981–0.989)	0.737 (0.671–0.803)	< 0.001
Random forest	0.969	0.668	0.958	0.667	0.980	0.668	0.980	0.113	0.959	0.969	0.969	0.194	0.996 (0.995–0.997)	0.730 (0.661–0.799)	< 0.001
Logistic regression	0.728	0.654	0.727	0.646	0.730	0.654	0.729	0.107	0.728	0.967	0.728	0.183	0.801 (0.786–0.815)	0.706 (0.626–0.786)	0.024
SVM	0.689	0.611	0.770	0.604	0.607	0.612	0.662	0.090	0.725	0.960	0.712	0.157	0.766 (0.751–0.782)	0.682 (0.598–0.765)	0.052
KNN	0.791	0.601	0.973	0.604	0.610	0.601	0.714	0.088	0.957	0.960	0.823	0.154	0.949 (0.942–0.955)	0.667 (0.585–0.749)	< 0.001
All-cause mortality															
MLP	0.770	0.741	0.816	0.716	0.724	0.745	0.747	0.291	0.797	0.947	0.780	0.414	0.836 (0.823–0.850)	0.796 (0.755–0.837)	0.065
Random forest	0.952	0.740	0.940	0.716	0.965	0.744	0.964	0.290	0.941	0.947	0.952	0.412	0.990 (0.988–0.993)	0.790 (0.750–0.831)	< 0.001
LightGBM	0.924	0.690	0.884	0.716	0.964	0.686	0.961	0.250	0.892	0.943	0.921	0.371	0.977 (0.972–0.981)	0.771 (0.725–0.816)	< 0.001
SVM	0.925	0.709	0.896	0.706	0.953	0.709	0.950	0.262	0.902	0.943	0.923	0.382	0.982 (0.978–0.985)	0.761 (0.714–0.808)	< 0.001
KNN	0.780	0.715	0.932	0.716	0.629	0.716	0.715	0.268	0.902	0.945	0.809	0.390	0.907 (0.897–0.917)	0.761 (0.713–0.808)	< 0.001
Logistic regression	0.751	0.666	0.770	0.667	0.731	0.666	0.741	0.226	0.761	0.932	0.755	0.337	0.812 (0.797–0.827)	0.760 (0.716–0.805)	0.031

*MLP* Multilayer perceptron, *LightGBM* Light Gradient Boosting Machine, *SVM* Support vector machine, *KNN* K-nearest neighbors, *ED* Emergency department, *PPV* Positive predictive value, *NPV* Negative predictive value, F1, 2 × (precision × recall/precision + recall), *AUC* Area under the curve, *CI* Confidence interval, *ICU* intensive care unit

^*^The DeLong test was used to compare the AUC between train and test models [27]

**Fig. 2:**
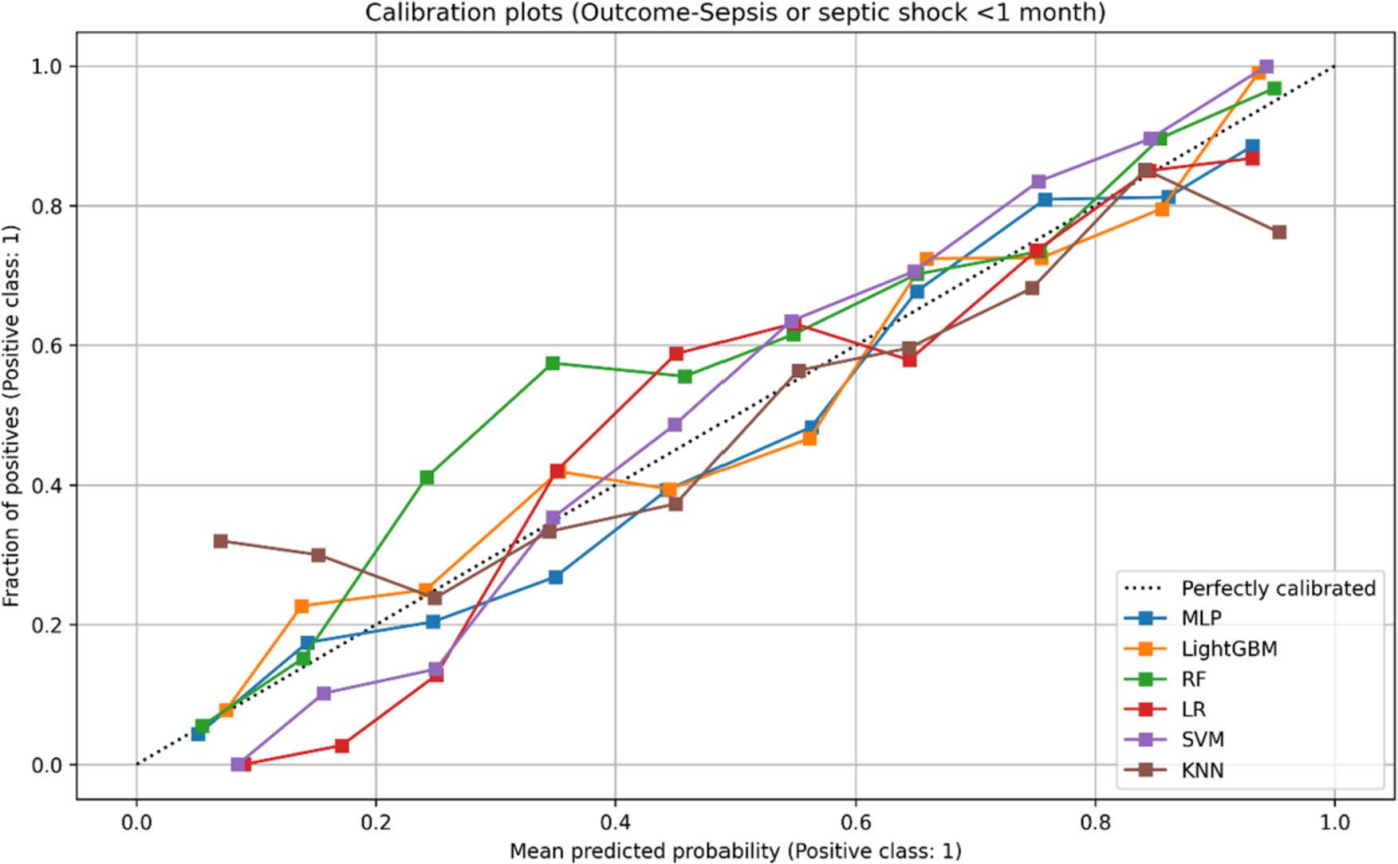
Calibration plot: predicted and true probability results for sepsis and septic shock

**Fig. 3:**
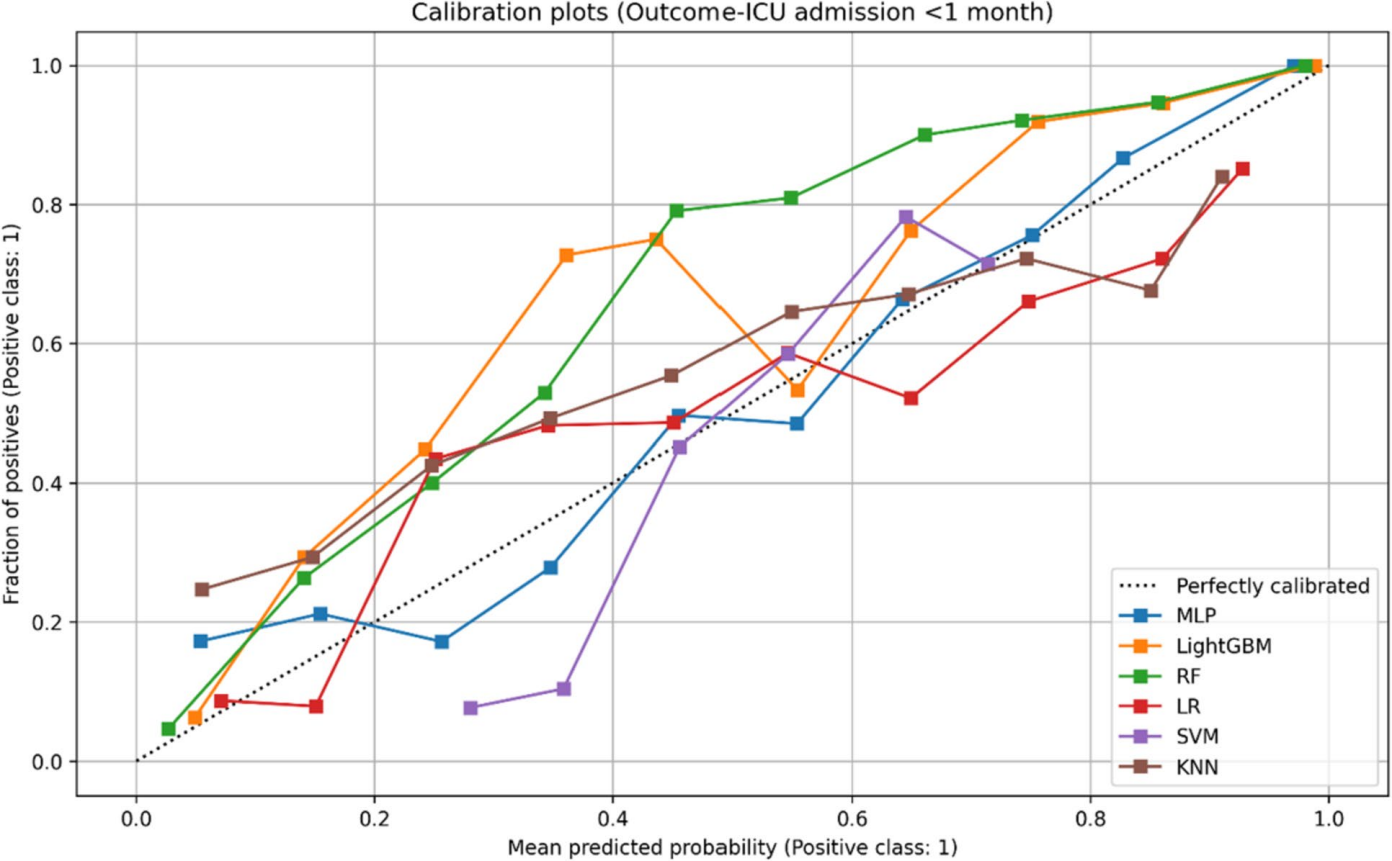
Calibration plot: predicted and true probability results for ICU admission. ICU, intensive care unit

**Fig. 4:**
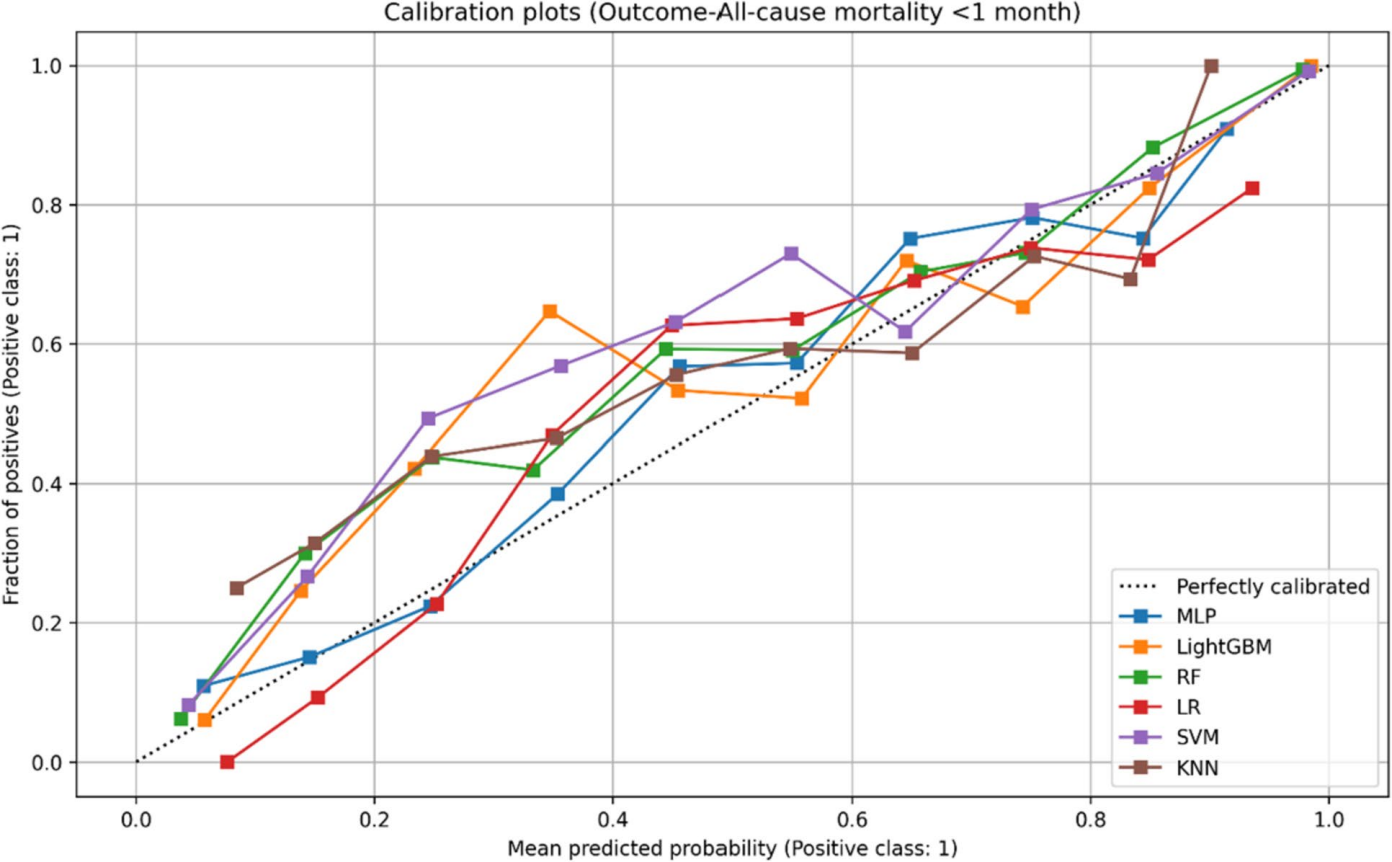
Calibration plot: predicted and true probability results for all-cause mortality

Meanwhile, it is crucial for models to be well calibrated when used in real-world patient-level scenarios, as inaccuracies in individual predicted probabilities may lead to inappropriate decisions by physicians. To assess the calibration of our models, we generated calibration plots that depict the distribution of observed and predicted case states across absolute probability subgroups or bins. A calibration curve that closely aligns with the diagonal indicates a higher level of calibration for the corresponding model. Our evaluation, as demonstrated in Figs. 2, 3 and 4, reveals that the calibration guideline for all MLP models was not significantly violated. Therefore, these models can be considered suitable for implementing a prediction system.

The HIS of the ED had an AI button (Supplementary Fig. 4) that displayed the prediction within 1 s after being pressed by the clinician (Supplementary Fig. 5). AI predictions were personalized and presented as percentages, with risks categorized as low (0%–33%), moderate (33%–66%), or high (66%–100%).

Patients with hyperglycemic crises (*n* = 271) between December 1, 2019 and April 30, 2021 were identified to compare the adverse outcomes between the non-AI and AI groups (Table 3). The AI group tended to have a lower ICU admission rate (11.1% vs. 19.8%) and all-cause mortality (11.1% vs. 15.0%) than the non-AI group; however, the differences were not significant. In addition, we used the same data to validate the PHD score and found that the AI model using MLP for predicting all-cause mortality performed better than the PHD score (Table 4).

**Table 3:** Comparison of clinical characteristics and adverse outcomes between the non-AI and AI groups in new ED patients with hyperglycemic crises between December 1, 2019 and April 30, 2021

Variable	Overall *n* = 271	Non-AI *n* = 253	AI *n* = 18	*p*-value
Age (years)	69.6 ± 16.6	70.0 ± 16.8	64.4 ± 13.5	0.113
Age subgroup (%)				0.090*
20 − 34	4.4	4.7	0	
35 − 49	8.1	7.5	16.7	
50 − 64	18.8	17.8	33.3	
65 − 74	21.4	20.9	27.8	
≥ 75	47.2	49.0	22.2	
Sex (%)				
Female	45.0	45.8	33.3	0.432
Male	55.0	54.2	66.7	
Body mass index (kg/m^2^)	22.7 ± 4.6	22.7 ± 4.6	22.9 ± 4.7	0.849
Asian BMI level subgroup (%)				
< 18.5	15.9	15.0	27.8	0.267
18.5 − 22.9	41.7	43.01	22.2	
23 − 24.9	17.0	17.0	16.7	
≥ 25	25.5	24.9	33.3	
Vital signs at triage				
Glasgow coma scale	11.7 ± 3.9	11.6 ± 3.9	13.67 ± 2.4	0.002
Systolic blood pressure (mmHg)	101.6 ± 23.0	102.3 ± 23.1	91.4 ± 19.8	0.037
Heart rate (beats/min)	137.8 ± 36.8	137.9 ± 36.1	135.9 ± 46.9	0.859
Respiratory rate (breaths/min)	20.1 ± 4.7	20.3 ± 4.7	18.0 ± 3.5	0.018
Body temperature (°C)	36.6 ± 0.9	36.6 ± 0.9	36.6 ± 0.5	0.893
Bedridden (%)	66.8	66.8	66.7	0.805
Nasogastric tub feeding (%)	14.0	14.2	11.1	> 0.999
Past histories (%)				
Hypertension	62.7	64.0	44.4	0.159
Hyperlipidemia	37.3	37.2	38.9	0.916
Cerebrovascular accident	33.6	34.8	16.7	0.189
Malignancy	15.1	15.4	11.1	> 0.999
Chronic kidney disease	22.9	22.9	22.2	> 0.999
Laboratory data				
Blood urea nitrogen (mg/dL)	28.2 ± 15.2	28.2 ± 15.7	28.8 ± 7.7	0.753
White blood cell count (10^3^/µL)	11.6 ± 6.4	11.6 ± 6.5	12.6 ± 5.5	0.440
Serum creatinine (mg/dL)	2.0 ± 1.7	2.0 ± 1.6	2.7 ± 2.3	0.234
Hemoglobin (g/dL)	12.5 ± 3.0	12.5 ± 3.0	13.0 ± 2.6	0.391
Glucose (mg/dL)	416.8 ± 367.3	407.7 ± 363.9	544.1 ± 401.1	0.177
hs-CRP (mg/L)	50.4 ± 83.8	51.9 ± 85.7	29.0 ± 47.4	0.076
Concomitant infection (%)	62.0	62.8	50.0	0.405
PHD score	2.4 ± 1.4	2.4 ± 1.4	2.4 ± 1.3	0.127
PHD risk class (%)				
Low risk (Score 0–2)	50.9	49.8	66.7	0.366
Intermediate risk (Score 3)	29.2	29.6	22.2	
High risk (Score ≥ 4)	19.9	20.6	11.1	
Outcomes < 1 month (%)				
Sepsis or septic shock	37.6	37.5	38.9	0.890
ICU admission	19.2	19.8	11.1	0.540
All-cause mortality	14.8	15.0	11.1	> 0.999

Data are presented as % or mean ± SD. The independent t-test was used to analyze continuous variables, while the Chi-Square test was utilized to examine categorical variables

*AI* Artificial intelligence, *ED* Emergency department, *ICU* Intensive care unit, *BMI* Body mass index, *hs-CRP* High sensitivity C-reactive protein, *PHD* Predicting the hyperglycemic crisis death, *SD* Standard deviation

^*^Because the number of an AI group in the age category “20–34” is 0, we only conducted the test for the other four age subgroups

**Table 4:** Comparison of predicting the ICU admission and all-cause mortality rates between the AI model using MLP and the PHD score

**All-cause mortality**	Accuracy	Sensitivity	Specificity	PPV	NPV	F1	AUC	*p*-value*
MLP model	0.776	0.637	0.797	0.314	0.938	0.421	0.796	< 0.001
PHD score	0.670	0.637	0.675	0.223	0.927	0.330	0.693	
**ICU admission**	Accuracy	Sensitivity	Specificity	PPV	NPV	F1	AUC	*p*-value
MLP model	0.809	0.521	0.827	0.161	0.964	0.246	0.743	0.084
PHD score	0.671	0.521	0.681	0.094	0.957	0.160	0.641	

*ICU* Intensive care unit, *AI* Artificial intelligence, *MLP* Multilayer perceptron, *PHD* Predicting the hyperglycemic crisis death, *PPV* Positive predictive value, *NPV* Negative predictive value; F1, 2 × (precision × recall/precision + recall), *AUC* Area under the curve

^*^The DeLong test was used to compare the AUC between MLP model and PHD score [27]. Note: We adjusted the classification threshold to approach the same level of sensitivity as the prediction using the PHD score

## Discussion

We developed an AI prediction model using MLP for ED patients with hyperglycemic crises that provided real-time decision-making assistance to physicians. The AUC of the model was 0.852 for sepsis or septic shock, 0.743 for ICU admissions, and 0.796 for all-cause mortality within 1 month. The impact study showed that the AI group tended to have lower ICU admissions and all-cause mortality than the non-AI group, but the differences were not significant.

Clinical decision rules (CDRs) like the PHD score can help with critical decision-making regarding patient health [37–39], but they have limitations. CDRs are designed to simplify complexity, and they should be externally validated in diverse settings to ensure applicability [37, 38]. They may not be applicable to a user’s clinical setting or a targeted population, and they require manual calculation, which can be inconvenient in a busy ED [37, 38].

AI is a breakthrough in healthcare that has the potential to improve the system. MLP, a significant model in the artificial neural network, is preferred for solving nonlinear problems. It consists of the input, hidden, and output layers and mimics the human brain [40]. Unlike other computerized tools, AI learns, tests, and generates autonomously by analyzing big data [23, 41]. AI offers various opportunities for ED care, including image interpretation, predicting patient outcomes, monitoring vital signs, reducing documentation burden with natural-language-processing, home monitoring systems, and outbreak prediction tools [41–44].

We integrated an AI prediction model into the HIS, which overcame barriers between AI research and clinical practice, but there were implementation barriers. Hospital policies and cooperation from the hospital information department were crucial for successful implementation. Additionally, incorporating AI into the HIS was technically challenging and may require overhauling existing information technology systems. Finally, concerns regarding malpractice, accuracy, and physician replacement by AI may affect physician acceptance of AI implementation [23].

Based on the same dataset, the AUC of all-cause mortality of the best model in our study was superior to that of the PHD score (0.796 vs. 0.693), suggesting that our AI model may be a better tool for predicting adverse outcomes in ED patients with hyperglycemic crises than the conventional PHD score.

We used the AUC, a recognized and comprehensive metric, to select the algorithm for our study [6, 9–11]. A major advantage of AUC is that it measures the ranking of predictions, rather than their absolute values, and is classification-threshold-invariant [45]. However, the choice of metric depends on the study’s aim [10]. For instance, if high sensitivity to predict sepsis or septic shock was the aim, we may have chosen LightGBM since it had the best sensitivity of 0.803 in our study.

We used the SHAP value, a new method to increase the transparency of AI prediction, to identify the importance of each feature variable for determining adverse outcomes [36]. In the SHAP summary plot, red and blue indicate high and low associations, respectively, between the feature variable and an adverse outcome [36].

The study implemented a real-time AI prediction model integrated in the HIS to predict adverse outcomes in ED patients with hyperglycemic crises, which was a major strength. However, there were some limitations. The AUC for predicting ICU admission was lower than that for sepsis or septic shock and all-cause mortality, possibly due to the subjective nature of ICU admission decision-making [46]. The results of the DeLong test (Table 2) indicate that, except for MLP models, there is a potential for overfitting in most models, which should be approached with caution. It is worth considering increasing the size of the data to potentially mitigate this issue and improve the performance of the models. The “black box” phenomenon remained a problem [23], but using the SHAP value may help increase transparency [36]. The impact of AI prediction on clinical practice was not fully evaluated, and further studies are needed. The AI prediction model may not be generalizable to other hospitals, and ethical and legislative issues may arise from using AI predictions as a tool. There were also limitations in the ICD measures [47, 48]. Lastly, the sample size of new patients was small, warranting more patients to be recruited to delineate this issue.

## Conclusions

We developed the first AI model to predict adverse outcomes in ED patients with hyperglycemic crises and integrated it into the HIS to provide real-time decision assistance. ED physicians obtained a second opinion from big data in real time using AI, which helped them in their decision making. The impact study showed no significant difference in the ICU admission or all-cause mortality rates between the non-AI and AI groups; however, further studies recruiting more patients will clarify this issue.

### Supplementary Information

**Additional file 1: Supplementary Table 1.** Hyper-parameters range for experiments. **Supplementary Table 2. **Statistics of missing value and given value for model training. **Supplementary Table 3.** The AI models for predicting ICU admission <48 hours in the ED patients with hyperglycemic crises. **Supplementary Table 4. **The *p*-value from the DeLong test to compare the model AUC.

**Additional file 2: Supplementary Figure 1. **Learning Curve for MLP in three adverse outcomes. **Supplementary Figure 2. **The AUC for three adverse outcomes in different algorithms. **Supplementary Figure 3. **SHAP values for the MP model. **Supplementary Figure 4.** AI button was integrated in the main screen of existing emergency department system. **Supplementary Figure 5.** A snapshot of the AI prediction result.

## Data Availability

The datasets analyzed for this study are available from the corresponding author upon reasonable request.

## Abbreviations

DKA: Diabetic ketoacidosis
HHS: Hyperosmolar hyperglycemic state
ED: Emergency department
ICU: Intensive care unit
PHD: Predicting the hyperglycemic crisis death
AUC: Area under the curve
AI: Artificial intelligence
CMMC: Chi Mei Medical Center
ICD-9-CM: International Classification of Diseases, Ninth Revision, Clinical Modification
BMI: Body mass index
GCS: Glasgow Coma Scale
hs-CRP: High-sensitivity C-reactive protein
EMRs: Electronic medical records
HIS: Hospital information system
ML: Machine learning
DL: Deep learning
SVM: Support vector machine
KNN: K-nearest neighbor
LightGBM: Light Gradient Boosting Machine
MLP: Multilayer perceptron
PPV: Positive predictive value
NPV: Negative predictive value
SHAP: SHapley Additive exPlanations
CDRs: Clinical decision rules
