# Supplementary material for: Using artificial intelligence to predict adverse outcomes in emergency department patients with hyperglycemic crises in real time
Source: BMC Endocr Disord. 2023 Oct 24;23:234. doi: 10.1186/s12902-023-01437-9 (PMC10594858; doi:10.1186/s12902-023-01437-9)
Supplement: Supplementary file 1 — Additional file 1: Supplementary Table 1. Hyper-parameters range for experiments. Supplementary Table 2. Statistics of missing value and given value for model training. Supplementary Table 3. The AI models for predicting ICU admission <48 hours in the ED patients with hyperglycemic crises. Supplementary Table 4. The p-value from the DeLong test to compare the model AUC. [file 12902_2023_1437_MOESM1_ESM.docx]

**Supplementary Table 1.** Hyper-parameters range for experiments

| Method and  Hyper-parameter |  | Outcomes -  Sepsis or septic shock |  | Outcomes -  ICU admission |  | Outcomes -  All-cause mortality |
| --- | --- | --- | --- | --- | --- | --- |
|  |  | Values |  | Values |  | Values |
| Logistic regression |  |  |  |  |  |  |
| penalty |  | l1, l2 |  | l1, l2 |  | l1, l2 |
| C |  | np.logspace(-3,3,7),  1, 5, 10 |  | np.logspace(-3,3,7),  1, 5, 10 |  | np.logspace(-3,3,7),  1, 5, 10 |
| max_iter |  | 7, 9, 10, 15, 50, 75, 100, 1000 |  | 7, 9, 10, 50, ,100,1000 |  | 7, 9, 10, 50, ,100,1000 |
| Defaults were used for other parameters |  | dual=False, tol=0.0001, fit_intercept=True, intercept_scaling=1, class_weight=None, random_state=None, solver='lbfgs', multi_class='auto', verbose=0, warm_start=False, n_jobs=None, l1_ratio=Non | | | | |
| Random forest |  |  |  |  |  |  |
| n_estimators |  | 110, 250, 500,  750, 950, 1000 |  | 110, 250, 250, 300,  500, 700, 1000 |  | 110, 250, 300,  500, 700, 950 |
| max_depth |  | 7, 9, 15, 30, 45, 50, 100 |  | 7, 9, 15, 30, 50, 90 |  | 7, 9, 15, 30, 50, 90 |
| min_samples_split |  | 2, 5 10, 15 |  | 2, 4, 6, 10, 15 |  | 2, 6, 10, 15 |
| max_features |  | auto, sqrt, 0.5,  1.0, 1.5, 2.5 |  | auto, sqrt, 0.5,  1.0, 1.5, 2.5 |  | auto, sqrt, 0.5,  1.0, 1.5, 2.5 |
| Defaults were used for other parameters |  | criterion='gini', min_samples_leaf=1, min_weight_fraction_leaf=0.0, max_leaf_nodes=None, min_impurity_decrease=0.0, bootstrap=True, oob_score=False, n_jobs=None, random_state=None, verbose=0, warm_start=False, class_weight=None, ccp_alpha=0.0, max_samples=None | | | | |
| KNN |  |  |  |  |  |  |
| n_neighbors |  | range(1,20) |  | range(1,20) |  | range(1,20) |
| weights |  | uniform, distance |  | uniform, distance |  | uniform, distance |
| algorithm |  | auto, ball_tree, kd_tree, brute |  | auto, ball_tree, kd_tree, brute |  | auto, ball_tree, kd_tree, brute |
| leaf_size |  | range(1,5) |  | range(1,5) |  | range(1,5) |
| Defaults were used for other parameters |  | p=2, metric='minkowski', metric_params=None, n_jobs=None | | | | |
| SVM |  |  |  |  |  |  |
| kernel |  | rbf, linear |  | rbf, linear |  | rbf, linear |
| gamma |  | scale, 1e-2, 1e-3, 1e-4 |  | scale, 1e-2, 1e-3, 1e-4 |  | scale, 1e-2, 1e-3, 1e-4 |
| C |  | 1, 10, 100, 500 |  | 1, 5, 10, 150, 250, 300 |  | 20, 50, 150, 200 |
| random_state |  | None, range(1,25,5) |  | None, range(1,25,5) |  | None, range(1,25,5) |
| decision_function_shape |  | ovo, ovr |  | ovo, ovr |  | ovo, ovr |
| shrinking |  | True, False |  | True, False |  | True, False |
| Defaults were used for other parameters |  | degree=3, coef0=0.0, probability=False, tol=0.001, cache_size=200, class_weight=None, verbose=False, max_iter=-1, break_ties=False | | | | |
| LightGBM |  |  |  |  |  |  |
| learning_rate |  | 1e-3, 1e-2 |  | 1e-4, 1e-3, 1e-2 |  | 1e-3, 1e-2 |
| n_estimators |  | 120, 200, 500, 750, 1000 |  | 120, 150, 300, 500, 750 |  | 120, 150, 250, 500, 750 |
| max_depth |  | 7, 9, 15, 30, 50 |  | 9, 15, 30, 50 |  | 9, 15, 30, 50 |
| random_state |  | 8, 16, 42 |  | 8, 16, 42 |  | 8, 16, 42 |
| Defaults were used for other parameters |  | boosting_type='gbdt', num_leaves=31, subsample_for_bin=200000, objective=None, class_weight=None, min_split_gain=0.0, min_child_weight=0.001, min_child_samples=20, subsample=1.0, subsample_freq=0, colsample_bytree=1.0, reg_alpha=0.0, reg_lambda=0.0, n_jobs=None, importance_type='split' | | | | |
| XGBoost |  |  |  |  |  |  |
| learning_rate |  | 1e-3, 1e-2, 1e-1 |  | 1e-3, 1e-2, 1e-1 |  | 1e-3, 1e-2, 1e-1 |
| gamma |  | 0, 1e-2, 1e-3, 1e-4, 1e-5 |  | 0, 1e-2, 1e-3, 1e-4 |  | 0, 1e-2, 1e-3, 1e-4 |
| n_estimators |  | 200, 500, 750, 900, 1000 |  | 100, 200, 250, 350, 500 |  | 150, 200, 350, 550 |
| max_depth |  | 3, 15, 25, 30, 50 |  | 3, 15, 30, 50 |  | 3, 15, 30, 50 |
| num_parallel_tree |  | 2, 5, 15 |  | 2, 5, 10 |  | 2, 5, 8, 10 |
| objective |  | binary:logistic |  | binary:logistic |  | binary:logistic |
| Defaults were used for other parameters |  | n_estimators: int = 100, verbosity=0, booster='gbtree', tree_method=’auto’, n_jobs=1, gamma=0, min_child_weight=1, max_delta_step=0, subsample=1, colsample_bytree=1, colsample_bylevel =1, colsample_bynode=1, reg_alpha=0, reg_lambda=0, scale_pos_weight=1, base_score=0.5, random_state=0, missing=None, monotone_constraints=None, interaction_constraints=None,  importance_type=None, gpu_id=None, validate_parameters=False, predictor='auto', enable_categorical=False | | | | |
| MLPClassifier |  |  |  |  |  |  |
| hidden_layer_sizes |  | (125,), (125, 35),  (100,75,30), (100,55)  (100,45), (100,),  (90,60), (90) |  | (125,), (125, 35),  (100,75,30), (100,55)  (100,45), (100,), (96,),  (90,60), (90) |  | (125,), (125, 35),  (100,75,30), (100,55)  (100,45), (100,), (95),  (90,60), (90) |
| max_iter |  | 1000, 500, 250,  200, 100, 50 |  | 1000, 500, 250,  200, 100, 50, 30 |  | 1000, 500, 250,  200, 100, 50, 30 |
| learning_rate_init |  | 1e-3, 1e-2, 1e-1 |  | 1e-3, 1e-2, 1e-1 |  | 1e-3, 1e-2, 1e-1 |
| early_stopping |  | True, False |  | True, False |  | True, False |
| alpha |  | 1e-4, 1e-5 |  | 1e-4, 1e-5 |  | 1e-4, 1e-5 |
| Defaults were used for other parameters |  | activation='relu', solver='adam', batch_size='auto', learning_rate='constant', power_t=0.5, shuffle=True, random_state=None, tol=0.0001, verbose=False, warm_start=False, momentum=0.9, nesterovs_momentum=True, validation_fraction=0.1, beta_1=0.9, beta_2=0.999, epsilon=1e-08, n_iter_no_change=10, max_fun=15000 | | | | |

The hyper-parameters that are not described in this table are set to the default values used in the scikit-learn library. Grid search with 5-fold cross-validation for hyper-parameters tuning for each algorithm was conducted for obtaining optimal model. ICU, intensive care unit; KNN, K-nearest neighbors; SVM, support vector machine; LightGBM, Light Gradient Boosting Machine; MLP, multilayer perceptron.

**Supplementary Table 2.** Statistics of missing value and given value for model training

| Variable | Number of missing values | % of total | Given value |
| --- | --- | --- | --- |
| Body mass index | 493 | 18.49% | Female mean: 23.31  Male mean: 23.62 |
| hs-CRP | 409 | 15.34% | 2.5 mg/dL |
| Blood urea nitrogen | 306 | 11.48% | 6 mg/dL |
| Glasgow coma scale | 254 | 9.53% | 15 |
| Systolic blood pressure | 49 | 1.84% | 130 mmHg |
| Serum creatinine | 28 | 1.05% | 0.84 mg/dL |
| Respiratory rate | 28 | 1.05% | 12 breaths/min |
| Heart rate | 23 | 0.86% | 70 beats/min |
| Glucose | 14 | 0.53% | 100 mg/dL |
| White blood cell count | 8 | 0.30% | 7x10^3^/µL |
| Hemoglobin | 8 | 0.30% | 12 g/dL |
| Body temperature | 7 | 0.26% | 37°C |

hs-CRP, high sensitivity C-reactive protein.

**Supplementary Table 3.** The AI models for predicting ICU admission <48 hours in the ED patients with hyperglycemic crises

| Outcomes and algorithms | Accuracy | | Sensitivity | | Specificity | | AUC | |
| --- | --- | --- | --- | --- | --- | --- | --- | --- |
|  | Train | Test | Train | Test | Train | Test | Train | Test |
| ICU admission <48 hours |  |  |  |  |  |  |  |  |
| MLP | 0.721 | 0.754 | 0.746 | 0.737 | 0.697 | 0.755 | 0.771 | 0.780 |
| Logistic regression | 0.760 | 0.655 | 0.746 | 0.684 | 0.775 | 0.654 | 0.840 | 0.767 |
| Random forest | 0.975 | 0.657 | 0.975 | 0.658 | 0.974 | 0.656 | 0.998 | 0.746 |
| LightGBM | 0.963 | 0.631 | 0.943 | 0.632 | 0.983 | 0.631 | 0.991 | 0.726 |
| SVM | 0.646 | 0.624 | 0.990 | 0.632 | 0.302 | 0.624 | 0.796 | 0.702 |
| KNN | 0.786 | 0.609 | 0.958 | 0.763 | 0.614 | 0.604 | 0.907 | 0.695 |

ICU, intensive care unit; ED, emergency department; MLP, multilayer perceptron; LightGBM, Light Gradient Boosting Machine; SVM, support vector machine; KNN, K-nearest neighbors.

**Supplementary Table 4.** The *p*-value from the DeLong test to compare the model AUC

| Sepsis or septic shock | MLP | RF | LightGBM | SVM | KNN | LR |
| --- | --- | --- | --- | --- | --- | --- |
| MLP | 1 | 0.540 | 0.235 | <0.001 | <0.001 | <0.001 |
| RF | 0.540 | 1 | 0.342 | <0.001 | 0.001 | 0.001 |
| LightGBM | 0.235 | 0.342 | 1 | 0.010 | 0.016 | 0.007 |
| SVM | <0.001 | <0.001 | 0.010 | 1 | 0.868 | 0.346 |
| KNN | <0.001 | 0.001 | 0.016 | 0.868 | 1 | 0.312 |
| LR | <0.001 | 0.001 | 0.007 | 0.346 | 0.312 | 1 |
| ICU admission | MLP | LightGBM | RF | LR | SVM | KNN |
| MLP | 1 | 0.890 | 0.747 | 0.294 | 0.133 | 0.075 |
| LightGBM | 0.890 | 1 | 0.776 | 0.423 | 0.181 | 0.069 |
| RF | 0.747 | 0.776 | 1 | 0.392 | 0.121 | 0.065 |
| LR | 0.294 | 0.423 | 0.392 | 1 | 0.145 | 0.172 |
| SVM | 0.133 | 0.181 | 0.121 | 0.145 | 1 | 0.671 |
| KNN | 0.075 | 0.069 | 0.065 | 0.172 | 0.671 | 1 |
| All-cause mortality | MLP | RF | LightGBM | SVM | KNN | LR |
| MLP | 1 | 0.661 | 0.154 | 0.070 | 0.031 | 0.003 |
| RF | 0.661 | 1 | 0.055 | 0.076 | 0.081 | 0.117 |
| LightGBM | 0.154 | 0.055 | 1 | 0.598 | 0.593 | 0.652 |
| SVM | 0.070 | 0.076 | 0.598 | 1 | 0.989 | 0.978 |
| KNN | 0.031 | 0.081 | 0.593 | 0.989 | 1 | 0.986 |
| LR | 0.003 | 0.117 | 0.652 | 0.978 | 0.986 | 1 |

AUC, area under the curve; RF, random forest; LR, logistic regression; LightGBM, Light Gradient Boosting Machine; SVM, support vector machine; KNN, K-nearest neighbors.
