# Supplementary material for: Using artificial intelligence to predict adverse outcomes in emergency department patients with hyperglycemic crises in real time
Source: BMC Endocr Disord. 2023 Oct 24;23:234. doi: 10.1186/s12902-023-01437-9 (PMC10594858; doi:10.1186/s12902-023-01437-9)

**Supplementary Figure 1.** Learning Curve for MLP in three adverse outcomes

| **Sepsis or septic shock**  **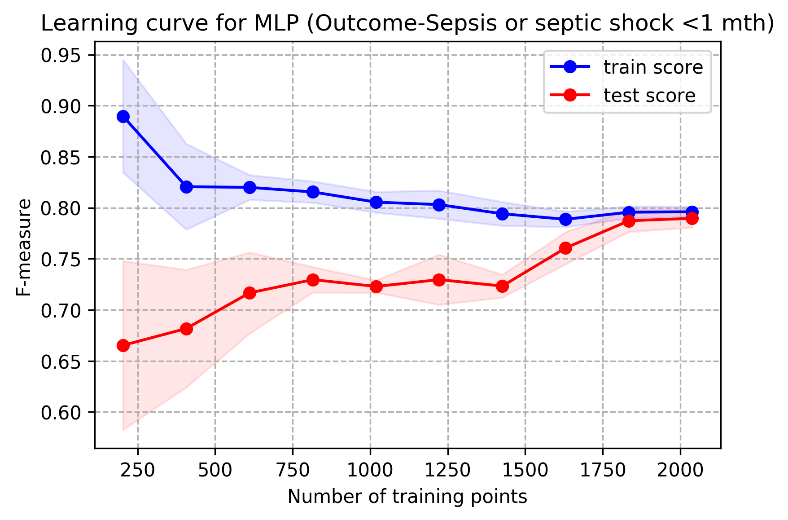** | **ICU admission**  **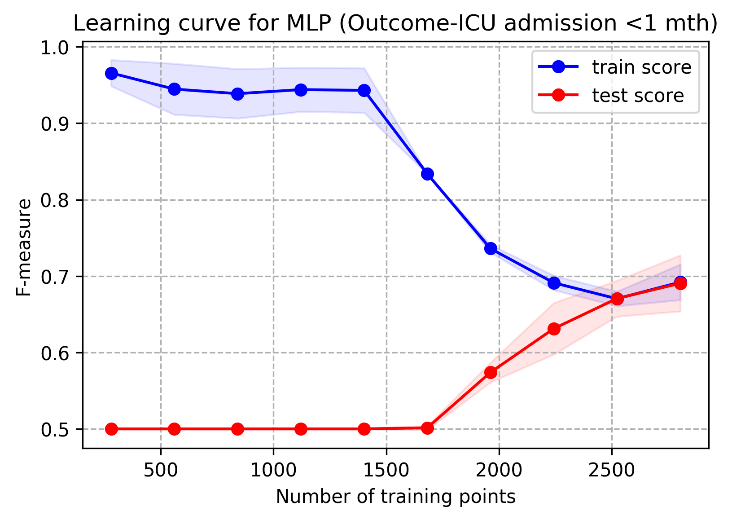** | **All-cause mortality**  **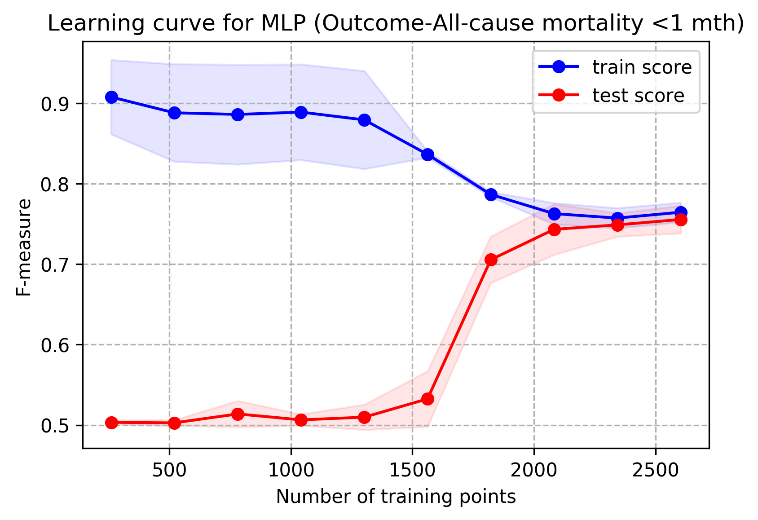** |
| --- | --- | --- |

**Supplementary Figure 2.** The AUC for three adverse outcomes in different algorithms

| **Sepsis or septic shock**  **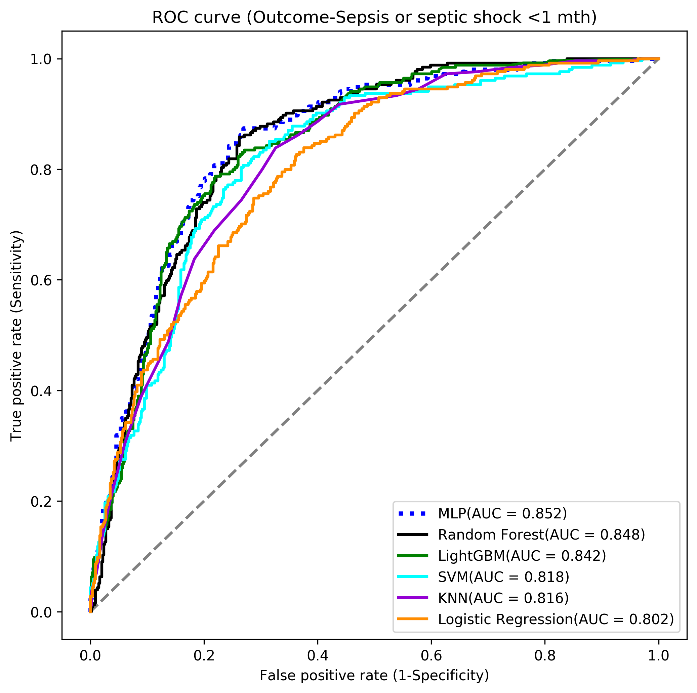** | **ICU admission**  **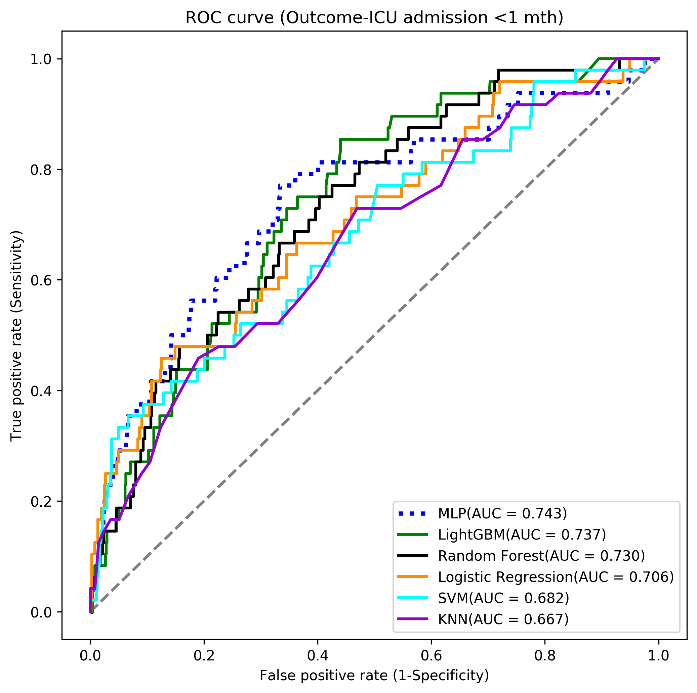** | **All-cause mortality**  **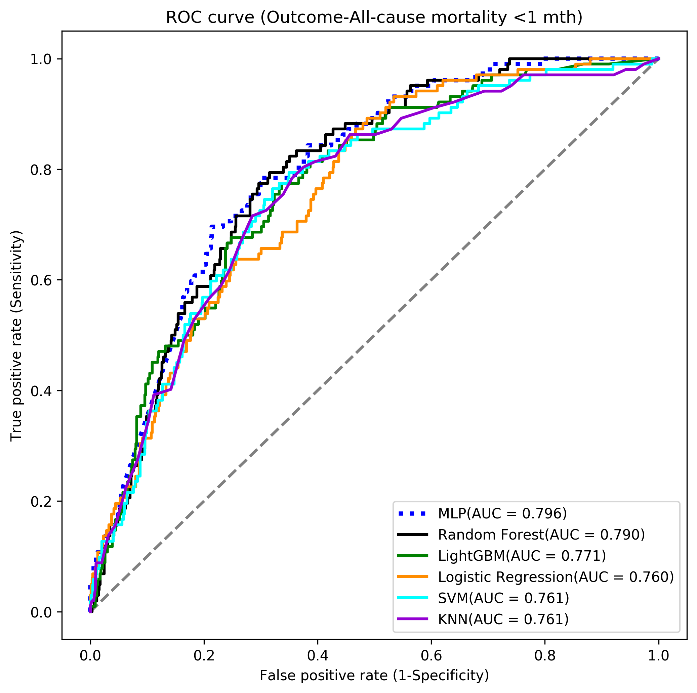** |
| --- | --- | --- |

**Supplementary Figure 3.** SHAP values for the MP model

**Sepsis and septic shock**

| a.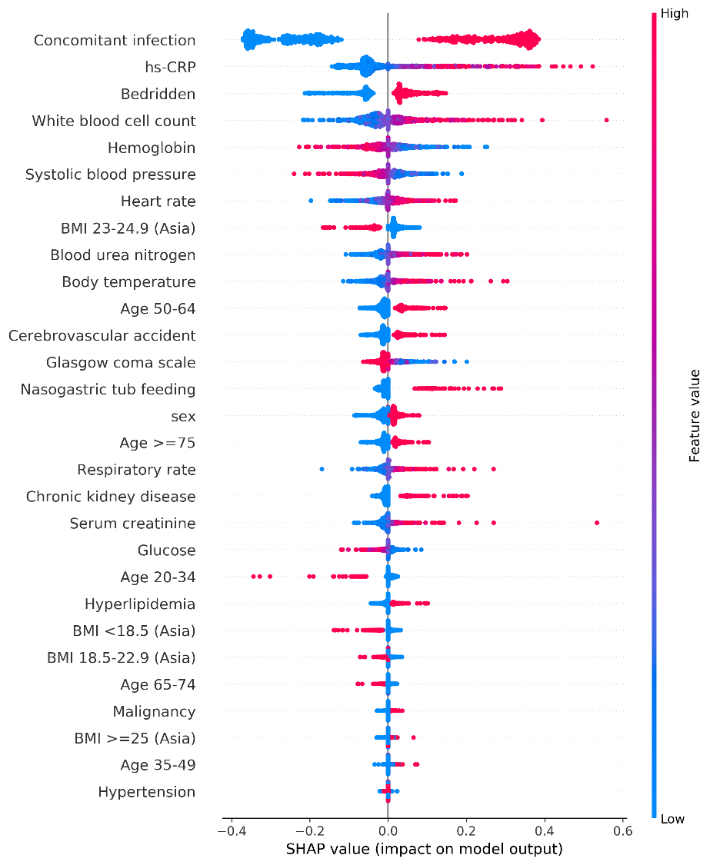 | b.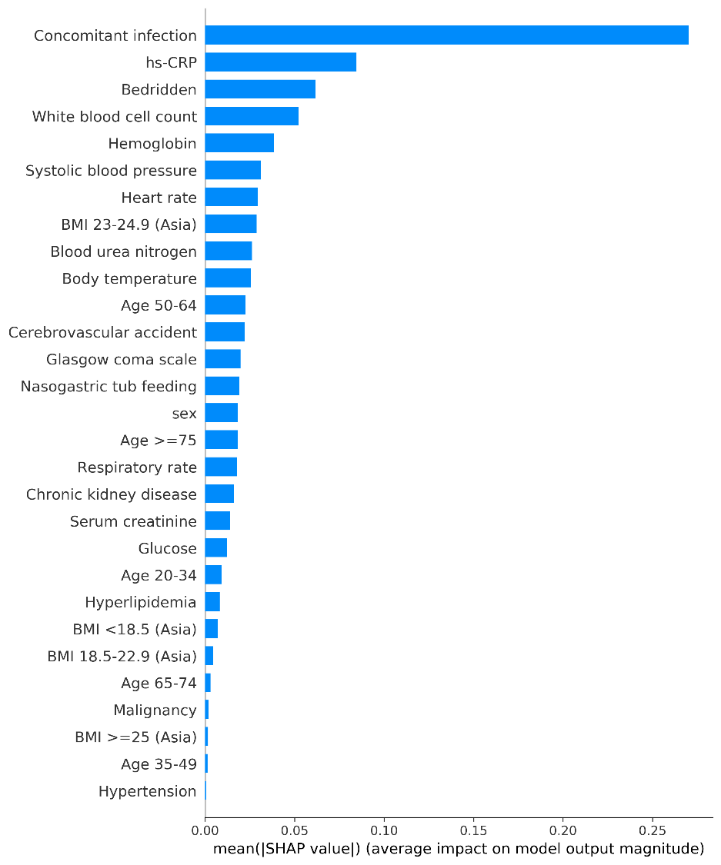 |
| --- | --- |

**ICU admission**

| a.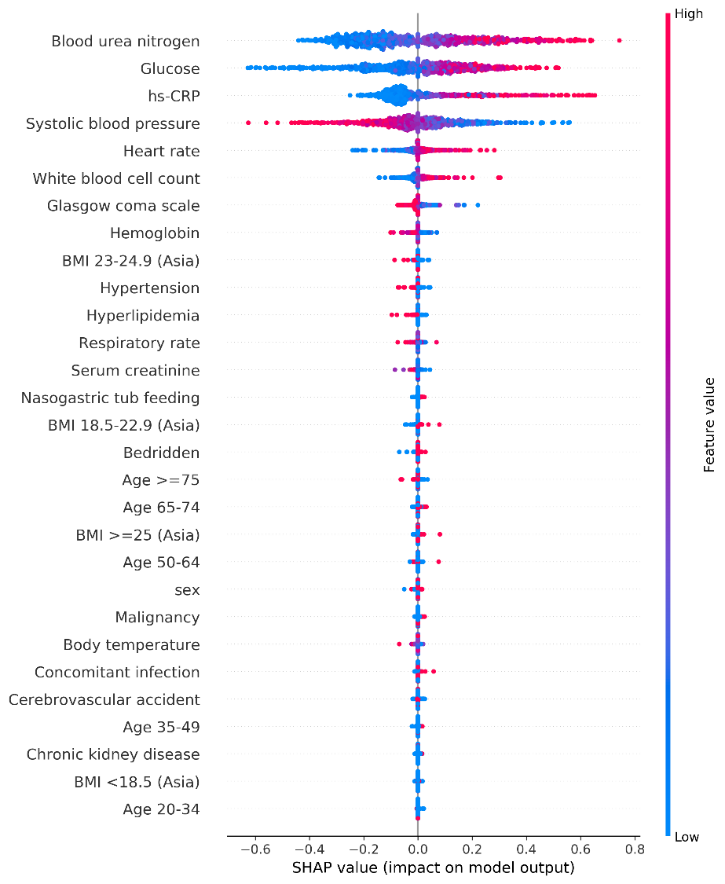 | b.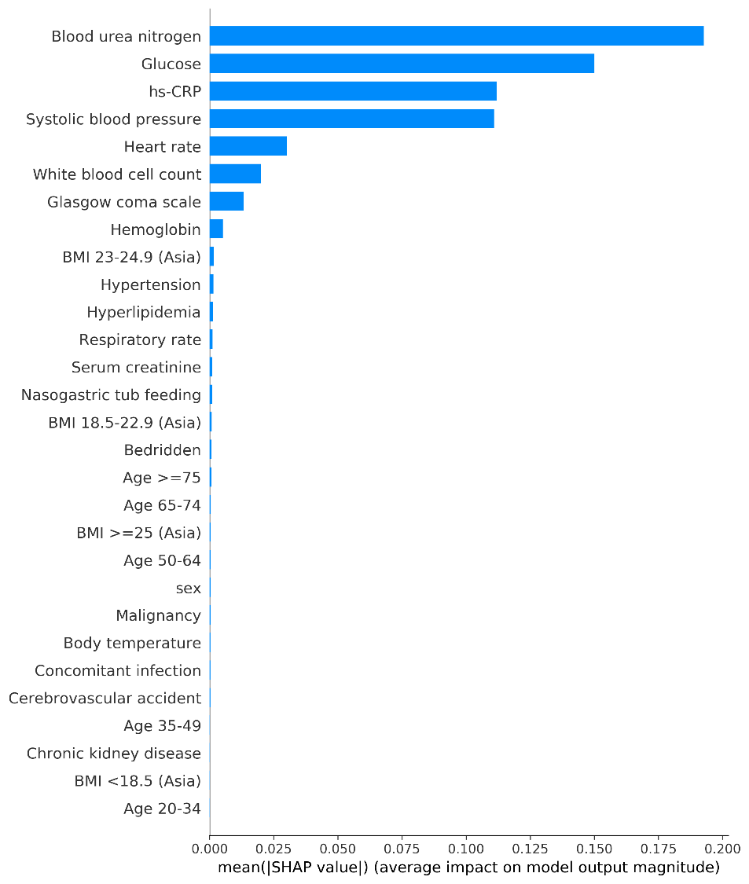 |
| --- | --- |

**All-cause mortality**

| a.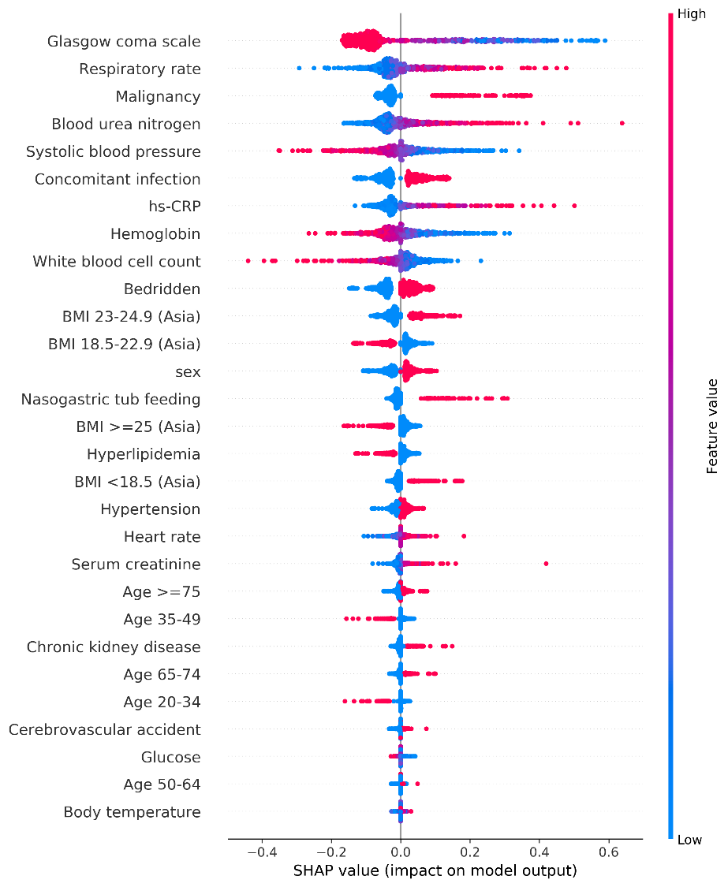 | b.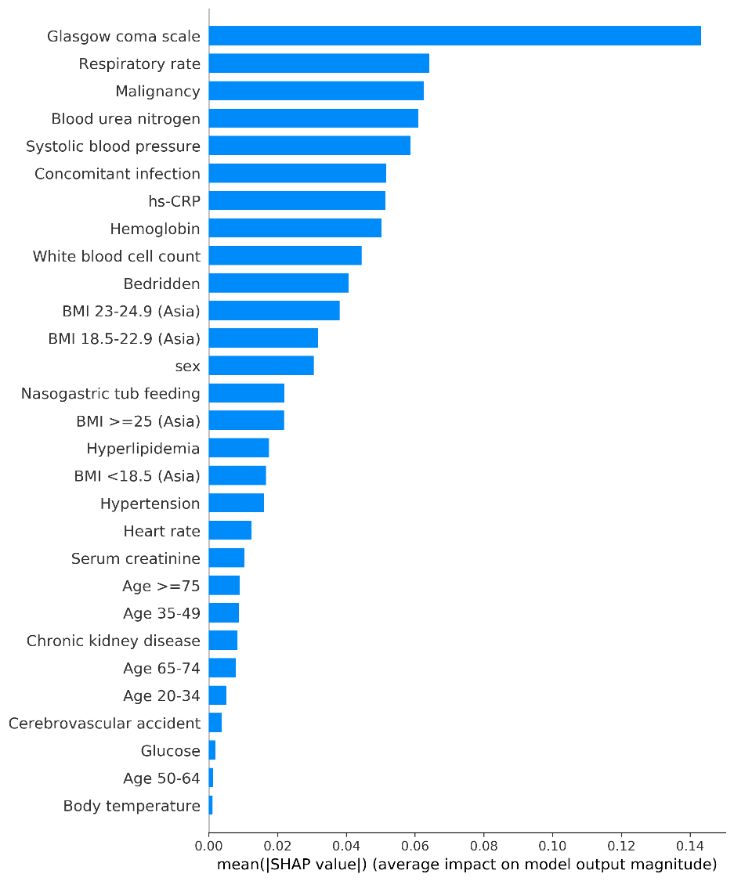 |
| --- | --- |

**Supplementary Figure 4.** AI button was integrated in the main screen of existing emergency department system


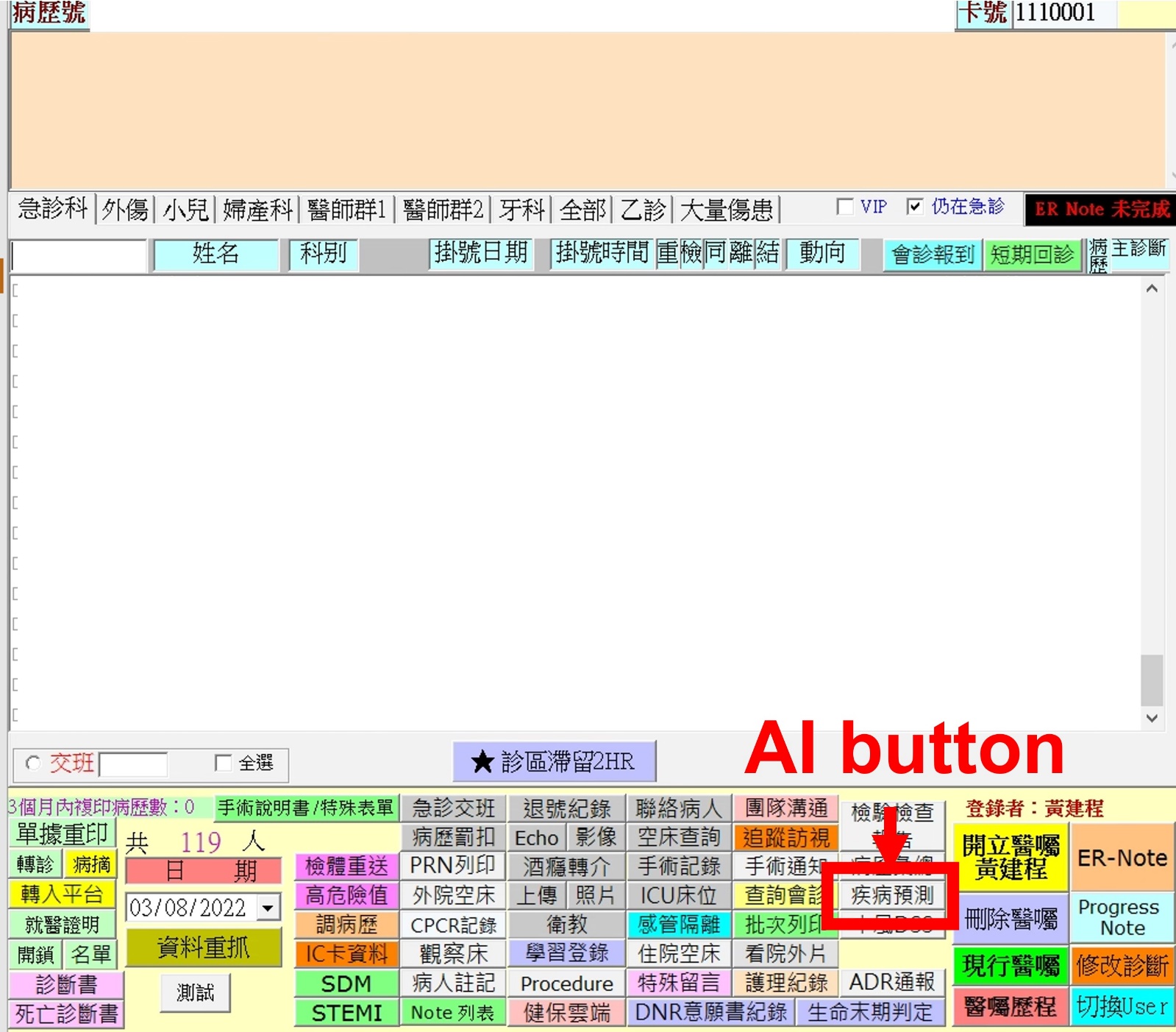


**Supplementary Figure 5.** A snapshot of the AI prediction result


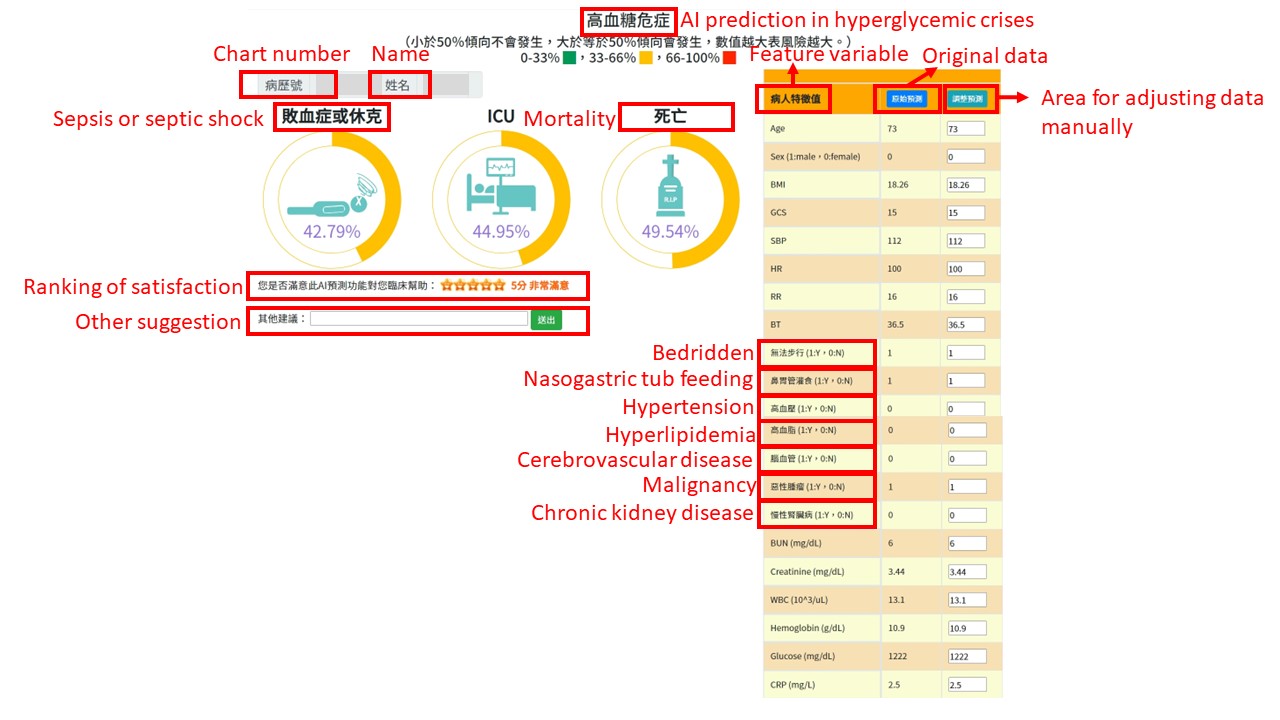

Supplement: Supplementary file 2 — Additional file 2: Supplementary Figure 1. Learning Curve for MLP in three adverse outcomes. Supplementary Figure 2. The AUC for three adverse outcomes in different algorithms. Supplementary Figure 3. SHAP values for the MP model. Supplementary Figure 4. AI button was integrated in the main screen of existing emergency department system. Supplementary Figure 5. A snapshot of the AI prediction result. [file 12902_2023_1437_MOESM2_ESM.docx]
